# Free Triiodothyronine Levels Are Associated with Diabetic Nephropathy in Euthyroid Patients with Type 2 Diabetes

**DOI:** 10.1155/2015/204893

**Published:** 2015-11-30

**Authors:** Jingcheng Wu, Xiaohua Li, Yang Tao, Yufei Wang, Yongde Peng

**Affiliations:** ^1^Department of Endocrinology and Metabolism, Shanghai First People's Hospital, School of Medicine, Shanghai Jiao Tong University, Shanghai 200080, China; ^2^School of Medicine, Jiangsu University, Zhenjiang, Jiangsu 212013, China; ^3^Department of Clinical Laboratory, Affiliated People's Hospital of Jiangsu University, Zhenjiang, Jiangsu 212002, China

## Abstract

*Objective*. To investigate the association of thyroid function and diabetic nephropathy (DN) in euthyroid patients with type 2 diabetes.* Methods*. A total of 421 patients were included in this cross-sectional study. The following parameters were assessed: anthropometric measurements, fast plasma glucose, serum creatinine, lipid profile, HbA1c, free triiodothyronine (FT3), free thyroxine, thyroid-stimulating hormone levels, and urinary albumin-to-creatinine ratio (UACR). Patients with UACR of ≥30 mg/g were defined as those suffering from DN.* Results*. Of the 421 patients, 203 (48.2%) suffered from DN, and no difference was found between males and females. The patients with DN yielded significantly lower FT3 levels than those without DN (*P* < 0.01). The prevalence of DN showed a significantly decreasing trend across the three tertiles based on FT3 levels (59.6%, 46.4%, and 38.6%, *P* < 0.01). After adjustment for gender and age, FT3 levels were found to correlate positively with estimated glomerular filtration rate (*P* = 0.03) and negatively with UACR (*P* < 0.01). Multiple linear regression analysis showed that FT3 level was independently associated with UACR (*β* = −0.18, *t* = −3.70, and *P* < 0.01).* Conclusion*. Serum FT3 levels are inversely associated with DN in euthyroid patients with type 2 diabetes, independent of traditional risk factors.

## 1. Introduction

Diabetic nephropathy (DN) is one of the most common microvascular complications of diabetes mellitus and leading causes of end-stage renal disease worldwide and accounts for a significant increase in morbidity and mortality in diabetic patients. Although DN is alleviated through conventional treatments, such as strict glycemic control, limited protein intake, blood pressure control, and renin-angiotensin-aldosterone system inhibition, these treatments cannot completely prevent the progression of DN in diabetic patients [1].

Several clinical studies show that thyroid dysfunction is related to renal disease. Overt and subclinical hypothyroidism are associated with a low estimated glomerular filtration rate (eGFR) and an increased risk of chronic kidney disease (CKD) [2–5]. These abnormalities are reversible, and improvement occurs after thyroid hormone replacement therapy is administered [2, 4]. Moreover, recent studies on euthyroid general population show that high normal levels of thyroid-stimulating hormone (TSH) and low normal levels of free triiodothyronine (FT3) are also associated with renal dysfunction, such as CKD and microalbuminuria [6–9].

Previous studies have shown a close interrelationship between thyroid hormone and DN. Type 2 diabetic patients with subclinical hypothyroidism (SCH) have been found to be associated with the high prevalence of DN in several studies [10, 11]. In 159 type 2 diabetic patients with euthyroidism and untreated SCH, the serum TSH level has been reported to be an independent risk factor of albuminuria [12]. In a recent study on patients with type 1 diabetes, Rodacki et al. [13] found that the prevalence of renal failure is higher not only in patients with SCH but also in those with high normal TSH levels, when compared with those with low normal TSH levels. This result suggests that the association between thyroid function and DN may extend into the euthyroid states. However, whether changes in normal thyroid function are related to DN in type 2 diabetic patients remains unknown. In the present research, a cross-sectional study was conducted to investigate the association of thyroid function with DN in euthyroid patients with type 2 diabetes.

## 2. Subjects and Methods

### 2.1. Study Population

A total of 632 patients with type 2 diabetes treated at the Shanghai Jiao Tong University Affiliated Shanghai First People's Hospital from 2009 to 2012 were enrolled in this study. The inclusion criteria were patients with normal thyroid function [0.25–4.2 mIU/L for TSH, 3.1–6.8 pmol/L for FT3, and 12.0–22.0 pmol/L for free thyroxine (FT4)] and negative for thyroid autoantibodies, such as thyroid peroxidase antibody (TPOAb) and thyrotropin receptor antibody (TRAb). The following exclusion criteria were considered: current malignancy, pregnancy, acute intercurrent illness, nondiabetic renal problems, chronic liver disease, urinary tract infection, history of thyroid disease, or any thyroid medication (levothyroxine or antithyroid drugs). A total of 421 patients (213 men and 208 women, mean age of 61.06 ± 10.35 years) were included in the final analysis. The study protocol was approved by the Ethics Committee of the Shanghai First People's Hospital.

### 2.2. Clinical and Laboratory Examination

Patient data, including demographic characteristics, lifestyle habits (smoking and drinking), medical history, and medication use, were obtained via a standard questionnaire. Smoking habit was defined as the consumption of more than five cigarettes daily for at least one year. Height and body weight were measured using a digital scale, and body mass index (BMI) was calculated by dividing body weight (in kilograms) by height (in square meters). Blood pressure (BP) was measured twice using a mercury sphygmomanometer, with participants in a seated position after 5 min of rest. Two BP readings were obtained 1 min apart, and the mean was calculated. Hypertension was defined as systolic blood pressure (SBP) of ≥140 mmHg and/or diastolic blood pressure (DBP) of ≥90 mmHg, or treatment with antihypertensive drugs.

Blood samples were collected after the patients fasted overnight. Biochemical parameters, including the levels of fasting plasma glucose (FPG), serum creatinine (SCr), serum total cholesterol (TC), serum triglyceride (TG), serum high-density lipoprotein cholesterol (HDL-C), and serum low-density lipoprotein cholesterol (LDL-C), were measured via routine laboratory methods by using a HITACHI 7600 instrument (HITACHI, Tokyo, Japan). HbA1c was detected through high-performance liquid chromatography (Hemoglobin Analyzer D-10, Bio-Rad Laboratories, Berkeley, USA). eGFR was calculated using the equation of the Modification of Diet in Renal Disease: eGFR (mL/min/1.73 m^2^) = 186 × (Scr/88.4)^−1.154^ × (age)^−0.203^ × (0.742 if female) [14].

Urinary albumin-to-creatinine ratio (UACR) was evaluated by obtaining the average of three UACR measurements. Before examination was conducted, the patients were instructed to avoid exercise for 1 h. Urinary albumin concentration was measured using immunoturbidimetry (Roche, Basel, Switzerland), and urinary creatinine concentration was determined by a modified Jaffe method on an automatic analyzer (Hitachi 7600, Tokyo, Japan). UACR was calculated as urinary albumin concentration divided by creatinine concentration and expressed in mg/g. DN was defined by an increased UACR of ≥30 mg/g in the absence of urinary tract infection or other renal abnormalities.

All of the patients were assessed by an experienced ophthalmologist using a direct ophthalmoscope and a digital retinal camera (Canon CR-DGi, Tokyo, Japan). Diabetic retinopathy (DR) was diagnosed according to the diagnostic code of the American Academy of Ophthalmology. The minimum criterion for the DR diagnosis was the presence of at least one microaneurysm in any photographed field [15].

Serums FT3, FT4, TSH, TPOAb, and TRAb levels were measured using an electrochemiluminescence analyzer (Cobas e601, Roche, Basel, Switzerland) with a mating reagent (the reference ranges of TSH, FT3, FT4, TPOAb, and TRAb were 0.25–4.2 mIU/L, 3.1–6.8, 12.0–22.0 pmol/L, <34 IU/mL, and <10 U/L, respectively).

### 2.3. Statistical Analysis

Continuous variables were expressed as mean ± standard deviation or median (interquartile range), and categorical variables were expressed as percentages. Continuous data were compared via Student's *t*-test or Mann-Whitney *U* test, and categorical data were compared via chi-square test. Non-normally distributed variables, such as HbA1c, TG, TSH, and UACR, were natural log-transformed or arctan-transformed into approximately normal distributed data before correlation and regression analysis were conducted. The correlation between serum thyroid hormone levels and related clinical variables was determined through partial correlation analysis. The independent determinants of UACR were identified through multiple linear regression analysis. A two-tailed *P* value of <0.05 was considered statistically significant. Data were analyzed using SPSS version 16.0 for Windows (SPSS Inc., Chicago, IL, USA).

## 3. Results

### 3.1. Clinical Characteristics of the Patients

Among the 421 patients, 203 (48.2%) were diagnosed with DN, and no difference was found between males and females (46.0% versus 50.5%, *P* > 0.05). The comparison results of the clinical characteristics between patients with and without DN are shown in Table 1. The patients with DN were older than those without DN. The patients with DN also experienced longer diabetic duration and exhibited higher BMI, SBP, and DBP, higher FPG, SCr, TG, and TC levels, and lower serum HDL-C and eGFR levels than those without DN. The patients with DN also showed a higher prevalence of hypertension and DR than those without DN. The former also used insulin, ACEI/ARBs, and statin/fibrates to a higher extent than the latter. Furthermore, the patients with DN yielded significantly lower FT3 levels than those without DN. The former also showed higher TSH levels and lower FT4 levels than the latter; however, the obtained values were not statistically significant.

### 3.2. Association of Thyroid Function with the Prevalence of DN

**Figure 1:**
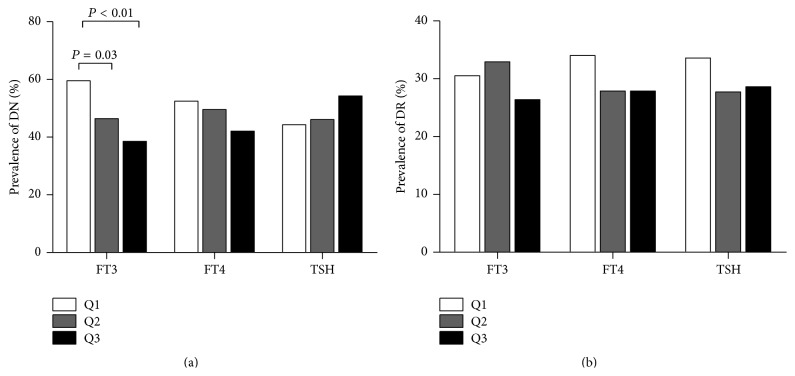
Prevalence of diabetic nephropathy (DN) (a) and diabetic retinopathy (DR) (b) among quartiles based on free triiodothyronine (FT3), free thyroxine (FT4), and thyroid-stimulating hormone (TSH) levels.

To investigate the association of thyroid function with DN, we divided the patients into three groups according to the tertiles of FT3 (<3.68, 3.68–4.09, and >4.09 pmol/L), FT4 (<16.03, 16.03–17.72, and >17.72 pmol/L), or TSH (<1.62, 1.62–2.40, and >2.40 mIU/L). The prevalence of DN showed a significantly decreasing trend across the three tertiles based on FT3 levels (59.6%, 46.4%, and 38.6%, *P* < 0.01 for the trend). The first FT3 quartile group showed a significantly higher prevalence of DN than the second and third groups (*P* = 0.03 and *P* < 0.01, resp.; Figure 1(a)). The prevalence of DN was not significantly different among the groups based on the tertiles of FT4 or TSH levels (Figure 1(a)). These results suggested that patients with low FT3 levels more likely develop DN than those with high FT3 levels. In our study, the prevalence of DR was not significantly different among FT3, FT4, and TSH tertiles (Figure 1(b)).

### 3.3. Relationship between Thyroid Function and Related Clinical Variables

The relationship between thyroid function and related clinical variables in all of the diabetic patients was examined through partial correlation analysis. After adjustment for gender and age, FT3 levels were correlated positively with TG and eGFR (*P* = 0.01 and *P* = 0.03, resp.); by contrast, FT3 levels were correlated negatively with FPG, HbA1c, and UACR (*P* = 0.04, *P* < 0.01, and *P* < 0.01, resp.). FT4 levels were also correlated negatively with HbA1c (*P* = 0.03). TSH levels were correlated positively with BMI and TG (*P* < 0.01 for both); conversely, TSH levels were correlated negatively with diabetic duration and HDL-C (*P* < 0.01 for both) (Table 2).

### 3.4. Analysis of Independent Variables Associated with UACR

The independent variables associated with UACR were assessed through multiple linear regression analysis; these variables included all the potential confounding factors in this study, such as gender, age, diabetic duration, smoking habit, BMI, hypertension, SBP, DBP, FPG, HbA1c, TG, TC, HDL-C, LDL-C, eGFR, TSH, FT3, FT4, presence or absence of DR, insulin use, ACEI/ARB intake, and statin/fibrate intake. We found that FT3 levels were independently associated with UACR (*β* = −0.18, *t* = −3.70, and *P* < 0.01). Other risk factors included diabetic duration, hypertension, DBP, presence or absence of DR, and FPG and eGFR levels (Table 3).

## 4. Discussion

In the present study, euthyroid patients with type 2 diabetes were investigated. Our results showed that the patients with DN yielded lower FT3 levels than those without DN. Moreover, the prevalence of DN decreased gradually as the quartiles of FT3 levels increased. Our results also indicated that FT3 levels were significantly correlated with UACR and eGFR levels. The results of our multiple linear regression analysis further revealed that FT3 levels were inversely associated with UACR after adjustment for a wide spectrum of lifestyle and biochemical risk factors. Therefore, FT3 levels possibly show a significant association with DN in euthyroid patients with type 2 diabetes.

Thyroid hormone plays an important role in the growth, development, and physiology of the kidneys. Thyroid dysfunction causes remarkable changes in renal blood flow, glomerular filtration rate, tubular secretory and absorptive capacity, electrolyte pumps, and kidney structure [16]. The results of previous studies suggest that overt and subclinical hypothyroidism are both associated with reduced eGFR and high prevalence of CKD and that these abnormalities can be normalized through thyroid hormone replacement therapy [2–5]. Moreover, thyroid hormone levels within the normal range are also found to be associated with the risk of CKD in the general population [6–8].

The relationship between the thyroid function and DN has also been reported by several previous studies. A study on 147 prediabetic subjects has shown that SCH is independently associated with the prevalence of microalbuminuria [17]. Two cross-sectional studies on type 2 diabetic patients have shown that SCH is independently associated with a high risk of DN [10, 11]. A small-scale study on type 2 diabetic patients with euthyroidism and untreated SCH has found an independent association between levels of serums TSH and UACR; however, whether or not this association still holds in euthyroid patients with type 2 diabetes is not mentioned in the study [12]. In a cross-sectional and multicentric study on patients with type 1 diabetes, Rodacki et al. [13] found that patients with low normal TSH levels (0.4–2.5 mIU/L) are associated with a lower risk of renal failure than patients with SCH (TSH ≥ 4.5 mIU/L) and high normal TSH levels (2.5–4.4 mIU/L). This result suggests that alterations of the thyroid function in the normal range are also associated with DN. However, as far as we know, no studies have reported the association between thyroid hormone levels and DN in euthyroid patients with type 2 diabetes.

The present study found that FT3 levels are inversely and independently associated with DN in euthyroid patients with type 2 diabetes. In agreement with our findings, those of Zhang et al. [8] demonstrate that low FT3 levels, even in the normal range, are moderately associated with an increased risk of incident CKD in a large cohort study on euthyroid individuals. Zhou et al. [9] also found that low serum FT3 levels in the normal range are associated with the high prevalence of microalbuminuria in middle-aged and elderly Chinese individuals. The above two studies both suggest that low normal FT3 levels are independently associated with kidney diseases. Nevertheless, the relationship between normal FT3 levels and DN has not been previously reported. In a previous study on type 2 diabetic patients with euthyroidism, FT3 levels are found to be correlated positively with eGFR; however, this study did not provide information on UACR levels of patients, and the association between FT3 levels and eGFR lost significance after adjustment for other metabolic factors [18]. A small-scale study has shown that total triiodothyronine (TT3) level is significantly correlated with urine microalbumin levels. Unfortunately, whether or not thyroid hormones are within the normal range remains unclear in this study; FT3 levels also have not been detected [19]. To the best of our knowledge, our study is the first to demonstrate that low FT3 levels are associated with DN in euthyroid patients with type 2 diabetes.

The mechanism of the association between FT3 and DN can be explained by the following. First, thyroid hormones elicit crucial effects on vascular and endothelial functions. Patients with SCH experience endothelial dysfunction characterized by a reduction in nitric oxide (NO) availability; this alteration is partially independent of dyslipidemia and can be reversed through levothyroxine supplementation [20]. Völzke et al. [21] found that serum TSH levels in the upper reference range are also associated with impaired endothelial function measured through flow-mediated dilation. In patients with advanced nondiabetic kidney disease, low T3 level is confirmed as a marker of endothelial dysfunction [22]. In experimental models, T3 influences endothelial function by inducing the relaxation of vascular smooth muscle cells through direct or indirect effects [23–25]. Cai et al. [26] also found that T3 can alleviate diabetic endothelial dysfunction in the arteries of diabetic rats. Endothelial dysfunction is associated with albuminuria in diabetes [27]; therefore, endothelial dysfunction is a possible link between low FT3 level and albuminuria. Second, Lin and Sun [28] found that T3 can attenuate albuminuria and improve renal structural damage in db/db diabetic mice by increasing phosphatidylinositol 3 kinase activity and by decreasing transforming growth factor-*β*1 expression. These two factors are implicated in DN progression [28]. Third, 3,5-diiodothyronine, a natural T3 metabolite via the deiodination pathway, can protect cells from renal damage in DN by inhibiting the activation of NF-*κ*B and JNK through enhancing of sirtuin 1 (SIRT1) expression [29]. NF-*κ*B and JNK pathways are also involved in the development of DN [30].

Interestingly, our study fails to show a significant association between TSH levels and albuminuria in diabetic patients, which is different from the results of previous studies. The possible explanations are as follows: Firstly, patients with positive TPOAb are not excluded from most previous studies. In general, patients with positive TPOAb have higher TSH levels than those without, even in the euthyroid state. Moreover, TPOAb is also found to be associated with renal disease. Xiang et al. [31] reported that endothelial dysfunction exists in Hashimoto's thyroiditis patients with euthyroidism and that TPOAb levels may be responsible for the endothelial dysfunction and subsequent microalbuminuria. Ara et al. [32] also found a high prevalence of antithyroid antibodies in patients with antiglomerular basement membrane antibody-mediated disease, which suggests a possible pathogenic link between TPOAb and renal disease. Therefore, these two findings both indicate that the presence of TPOAb should be adjusted in the analysis of the association between high TSH and albuminuria. In the present study, we exclude patients with positive TPOAb to avoid this bias. Secondly, metformin, a common antidiabetic drug, can induce a reduction in TSH levels in patients with type 2 diabetes [33, 34]. This finding suggests that TSH may be not* a relevant* parameter in assessing the thyroid function in diabetes.

Similar to other studies [35, 36], the present study found that thyroid function is not associated with DR; thus, the protective function of FT3 in diabetic patients may be organ specific. In our study, FT3 levels were correlated positively with TG; by contrast, FT3 levels were correlated negatively with FPG and HbA1c in euthyroid patients with type 2 diabetes. These results are consistent with those in a previous study [18]. The FT3 level (at the upper limit of the normal range) of overweight and obese patients increases compared with that of subjects with normal weight [37, 38]; this finding provided a possible explanation for the positive relationship between FT3 and TG levels. In in vitro studies, T3 protects cells from apoptosis and induces *β*-cell growth and proliferation in human and rodent insulinoma cell lines [39]. A clinical study has also demonstrated that FT3 may stimulate insulin secretion in euthyroid individuals with normal glucose tolerance [40]. These observations may account for the negative correlation of FT3 levels with FPG and HbA1c in our study.

As a limitation, a cross-sectional design was implemented in our study. This cross-sectional study investigated patients with type 2 diabetes treated with various medications. Further prospective and longitudinal studies should be conducted to confirm a causal relationship between FT3 levels and DN in diabetic patients, who are stratified with and without medications.

## 5. Conclusion

In summary, our study suggests that serum FT3 levels are inversely associated with DN in euthyroid patients with type 2 diabetes, which is independent of other risk factors. The results of our study may help predict the risk of DN development. Our study may also improve our understanding of this disease. Furthermore, our study can be used as a basis to establish effective prevention strategies.

## Figures and Tables

**Table 1:** Comparison of clinical characteristics between DN groups.

	Total (*n* = 421)	Patients without DN (*n* = 218)	Patients with DN (*n* = 203)	*P*
Age (y)	61.06 ± 10.35	59.59 ± 10.03	62.64 ± 10.48	<0.01
Gender (male/female)	213/208	115/103	98/105	0.36
Diabetic duration (years)	9.67 ± 7.45	8.57 ± 7.52	10.85 ± 7.21	<0.01
Smoking habit, *n* (%)	142 (33.7)	83 (38.1)	59 (29.1)	0.06
BMI (kg/m^2^)	24.92 ± 3.23	24.50 ± 2.99	25.37 ± 3.41	<0.01
Hypertension, *n* (%)	244 (58.0)	100 (45.9)	144 (70.9)	<0.01
SBP (mmHg)	133.03 ± 15.86	129.99 ± 15.49	136.29 ± 15.61	<0.01
DBP (mmHg)	79.66 ± 9.10	77.84 ± 8.97	81.67 ± 8.84	<0.01
FPG (mmol/L)	8.50 ± 2.73	8.15 ± 2.56	8.88 ± 2.86	<0.01
HbA1c (%)	8.8 (7.4–10.3)	8.7 (7.3–10.3)	8.9 (7.5–10.1)	0.70
SCr (*μ*mol/L)	60.0 (48.5–72.0)	57.0 (48.0–68.0)	63.0 (50.0–77.0)	<0.01
TG (mmol/L)	1.55 (1.13–2.32)	1.41 (1.04–2.02)	1.81 (1.25–2.76)	<0.01
TC (mmol/L)	4.92 ± 1.00	4.85 ± 0.92	5.00 ± 1.08	<0.01
HDL-C (mmol/L)	1.10 ± 0.24	1.13 ± 0.24	1.07 ± 0.24	<0.01
LDL-C (mmol/L)	2.86 ± 0.80	2.83 ± 0.77	2.89 ± 0.84	0.46
UACR (mg/g)	29.0 (16.0–75.0)	16.0 (11.0–23.0)	80.0 (48.0–154.0)	<0.01
eGFR (mL/min·1.73 m^2^)	115.40 ± 35.86	125.20 ± 33.25	104.79 ± 35.63	<0.01
Insulin, *n* (%)	133 (31.6)	57 (26.1)	76 (37.4)	0.01
ACEI/ARB, *n* (%)	83 (19.7)	28 (12.8)	55 (27.1)	<0.01
Statins/fibrates, *n* (%)	104 (24.7)	38 (17.4)	66 (32.5)	<0.01
DR, *n* (%)	126 (29.9)	47 (21.6)	79 (38.9)	<0.01
TSH (mIU/L)	1.96 (1.38–2.74)	1.87 (1.35–2.73)	2.14 (1.40–2.74)	0.10
FT3 (pmol/L)	3.88 ± 0.42	3.95 ± 0.41	3.81 ± 0.42	<0.01
FT4 (pmol/L)	16.88 ± 2.34	16.96 ± 2.41	16.81 ± 2.25	0.52

Data are expressed as means ± SD, median (interquartile range), or numbers (percentages).

DN, diabetic nephropathy; BMI, body mass index; SBP, systolic blood pressure; DBP, diastolic blood pressure; FPG, fasting plasma glucose; SCr, serum creatinine; TG, triglyceride; TC, total cholesterol; HDL-C, high-density lipoprotein cholesterol; LDL-C, low-density lipoprotein cholesterol; UACR, urinary albumin-to-creatinine ratio; eGFR, estimated glomerular filtration rate; DR, diabetic retinopathy; TSH, thyroid-stimulating hormone; FT3, free triiodothyronine; FT4, free thyroxine.

**Table 2:** Relationship between thyroid function and related clinical variables.

	FT3		FT4		TSH	
	*r*	*P* ^*∗*^	*r*	*P* ^*∗*^	*r*	*P* ^*∗*^
Diabetic duration	0.01	0.90	−0.03	0.48	−0.16	<0.01
BMI	0.02	0.64	0.07	0.17	0.16	<0.01
SBP	0.01	0.89	0.03	0.62	0.04	0.46
DBP	−0.03	0.50	0.06	0.24	0.09	0.08
FPG	−0.10	0.04	−0.09	0.07	−0.01	0.82
HbA1c	−0.14	<0.01	−0.11	0.03	−0.03	0.55
TG	0.13	0.01	0.08	0.11	0.19	<0.01
TC	0.06	0.26	0.06	0.21	0.07	0.17
HDL-C	0.04	0.46	−0.03	0.61	−0.13	<0.01
LDL-C	−0.02	0.71	0.05	0.32	0.04	0.47
UACR	−0.19	<0.01	−0.02	0.65	0.06	0.25
eGFR	0.10	0.03	0.04	0.38	−0.03	0.55

Data for TSH, HbA1c, and UACR levels were natural log-transformed, and those for TG levels were arctan-transformed before analysis. ^*∗*^ *P* values were adjusted for age and gender.

BMI, body mass index; SBP, systolic blood pressure; DBP, diastolic blood pressure; FPG, fasting plasma glucose; TG, triglyceride; TC, total cholesterol; HDL-C, high-density lipoprotein cholesterol; LDL-C, low-density lipoprotein cholesterol; UACR, urinary albumin-to-creatinine ratio; eGFR, estimated glomerular filtration rate; FT3, free triiodothyronine; FT4, free thyroxine; TSH, thyroid-stimulating hormone.

**Table 3:** Multiple linear regression analysis of independent variables associated with UACR.

	*β*	*t*	*P*
Diabetic duration	0.11	2.05	0.04
Hypertension	0.12	2.06	0.04
DBP	0.13	2.23	0.02
FPG	0.13	2.20	0.03
FT3	−0.18	−3.70	<0.01
eGFR	−0.25	−5.11	<0.01
Presence of DR	0.21	4.40	<0.01

Data for TSH, HbA1c, and UACR levels were natural log-transformed, and those for TG levels were arctan-transformed before analysis.

UACR, urinary albumin-to-creatinine ratio; DBP, diastolic blood pressure; FPG, fasting plasma glucose; FT3, free triiodothyronine; eGFR, estimated glomerular filtration rate; DR, diabetic retinopathy.
